# DNA methylation and single nucleotide variants in the brain-derived neurotrophic factor (BDNF) and oxytocin receptor (OXTR) genes are associated with anxiety/depression in older women

**DOI:** 10.3389/fgene.2015.00230

**Published:** 2015-06-30

**Authors:** Yvon C. Chagnon, Olivier Potvin, Carol Hudon, Michel Préville

**Affiliations:** ^1^Department of Psychiatry and Neurosciences, Laval University, Quebec CityQC, Canada; ^2^Research Center: Institut Universitaire en Sante Mentale de Quebec, Quebec CityQC, Canada; ^3^School of Psychology, Laval University, Quebec CityQC, Canada; ^4^Department of Sciences de la Santé Communautaire, Sherbrooke University, SherbrookeQC, Canada

**Keywords:** methylation, aging, anxiety, snps, BDNF Val66Met, OXTR

## Abstract

**Background:** Environmental effects and personal experiences could be expressed in individuals through epigenetic non-structural changes such as DNA methylation. This methylation could up- regulate or down-regulate corresponding gene expressions and modify related phenotypes. DNA methylation increases with aging and could be related to the late expression of some forms of mental disease. The objective of this study was to evaluate the association between anxiety disorders and/or depression in older women and DNA methylation for four genes related to anxiety or depression.

**Methods**: Women aged 65 and older with (*n* = 19) or without (*n* = 24) anxiety disorders and/or major depressive episode (DSM-IV), were recruited. DNA methylation and single nucleotide variant (SNV) were evaluated from saliva, respectively by pyrosequencing and by PCR, for the following genes: brain-derived neurotrophic factor (BDNF; rs6265), oxytocin receptor (OXTR; rs53576), serotonin transporter (SLC6A4; rs25531), and apolipoprotein E (APOE; rs429358 and rs7412).

**Results**: A greater BDNF DNA methylation was observed in subjects with anxiety/depression compared to control group subjects (Mean: 2.92 SD ± 0.74 vs. 2.34 ± 0.42; *p*= 0.0026). This difference was more pronounced in subjects carrying the BDNF rs6265 CT genotype (2.99 ± 0.41 vs. 2.27 ± 0.26; *p*= 0.0006) than those carrying the CC genotype (*p*= 0.0332); no subjects with the TT genotype were observed. For OXTR, a greater DNA methylation was observed in subjects with anxiety/depression, but only for those carrying the AA genotype of the OXTR rs53576 SNV, more particularly at one out of the seven CpGs studied (7.01 ± 0.94 vs. 4.44 ± 1.11; *p*= 0.0063). No significant differences were observed for APOE and SLC6A4.

**Conclusion**: These results suggest that DNA methylation in interaction with SNV variations in BDNF and OXTR, are associated with the occurrence of anxiety/depression in older women.

## Introduction

It is well-known that many mental diseases include a significant hereditary component. For instance, the heritability of anxiety disorders and depression, which are among the most prevalent and costly psychiatric disorders (Rice and Miller, 1998; Kessler et al., 2005; Olesen et al., 2012), is estimated at between 20 and 40% (Scherrer et al., 2000; Sullivan et al., 2000; Hettema et al., 2001; Kendler et al., 2001). Children of individuals with anxiety disorders show a significant increase risk (4–6 times more) of developing the same anxiety disorder as their parents in contrast to children without affected parents. Therefore the major source of this familial risk is genetic, at least according to the greater co-occurrence of anxiety disorders in identical monozygotic twins compared to non-identical dizygotic twins (Hettema et al., 2001). And, finally, anxiety disorders and depressive disorders are highly comorbid (Beekman et al., 2000; Kessler et al., 2003, 2010), and previous results suggest a notable mutual genetic origin (Neale and Kendler, 1995; Roy et al., 1995).

In contrast with autosomal dominant genetic disorder, such as Huntington’s disease where defect of a single gene is responsible of the pathology, anxiety disorders and depression are viewed as complex traits. They include a hereditary portion with multiple genetic anomalies within several different genes interacting together, as well as with environmental factors. Among genes involved in anxiety and depression, the gene which has received most attention is probably the serotonin transporter (SLC6A4 previously 5-HTT). SLC6A4 promotor showed a well-studied length polymorphism (5-HTTLPR) with a short or a long form. The short form has been associated with a reduced transcriptional efficiency of SLC6A4, resulting in decrease SLC6A4 expression and serotonin reuptake compared to the long form (Lesch et al., 1996; Gerretsen and Pollock, 2008). The short form has also been associated with the presence of social phobia (Furmark et al., 2004), obsessive-compulsive disorder (Hasler et al., 2006), post-traumatic stress disorder and depression (Kilpatrick et al., 2007), and with anxiety-related traits such as neuroticism and danger avoidance (Lesch et al., 1996). However, a meta-analysis did not confirm the association of the short form with depression (Risch et al., 2009).

Among other genes, the brain-derived neurotrophic factor (BDNF) has been associated with major depression (Verhagen et al., 2010; Pei et al., 2012) and with neuroticism (Frustaci et al., 2008). Lower BDNF level was observed in individuals with depression (Brunoni et al., 2008) and in post-mortem brain of suicide victims with major depression (40%) or other psychiatric disorders (Dwivedi et al., 2003; Karege et al., 2005). Moreover, a higher level of BDNF has been observed in those Alzheimer’s patients showing symptoms of depression compared to those with no depressive symptoms (Hall et al., 2011). Data from individuals with depression suggest that a substitution from valine to methionine, at amino-acid position 66 (BDNF Val66Met), could be responsible for structural alterations in the hippocampus and the prefrontal cortex (Cardoner et al., 2013) and in the uncinate fasciculus, a fiber tract linking these two regions (Carballedo et al., 2012). It has been proposed that these neural alterations in patients with depression, could result in poorer treatment outcomes (Cardoner et al., 2013).

The oxytocin receptor (OXTR) is another gene that appears to be a factor in the etiology of anxiety and depression. The haplotype GGGTGTC of the SNVs OXTR rs11131149, rs2243370, rs2243369, rs13316193, rs2254298, rs2268493, and rs2268491 was associated with depressive temperament (Kawamura et al., 2010), while in female adolescents, with adverse parental environment, anxiety and depressive symptoms were the most severe in those heterozygous for OXTR rs2254298 (Thompson et al., 2011). It was also shown that carriers of the GG and AG genotypes of the SNV OXTR rs53576 may present a greater biological sensitivity as well as stress reactivity in terms of environmental adaptation (Chang et al., 2014).

Finally, the apolipoprotein E (APOE) is a well-studied gene in geriatric psychiatry with three major forms of APOE resulting from the combination of two functional SNVs changing the protein amino acids, and named: APOE2 (Cys112, Cys 158), APOE3 (Cys112, Arg158), and APOE4 (Arg112, Arg158; Mahley and Rall, 2000). APOE3 is the most frequent one and is observed in about 78% of the population, while APOE2 and APOE4 are observed in about 6 and 17% of the population, respectively (Eisenberg et al., 2010). Unlike APOE2 and APOE3, APOE4 increases the risk of Alzheimer’s diseases (Poirier et al., 1993) and vascular dementia associated with a higher risk of cognitive decline (Lavretsky et al., 2003; Corsentino et al., 2009; Niti et al., 2009). Additionally it has been proposed that APOE is implicated in anxiety (Raber, 2007; Siegel et al., 2012), but data in humans on this topic are scarce. Data on female mice indicate that the females with APOE4 allele had higher anxiety level than the carriers of the APO2 and APOE3 alleles (Siegel et al., 2012).

DNA methylation is the main studied epigenetic factor modulating gene effects. Following an environmental stimulus, a methyl group could be added by methylase enzymes to the cytosine (C), located just before a guanine (G) in CpG DNA regions. This could be reversed by demethylase enzymes and/or be heritably transmitted to descents. It was observed that with aging some DNA regions rich in CpG become hypermethylated, whereas those DNA regions poor in CpG could become hypomethylated (Heyn et al., 2012). For instance, variations in methylation showed a strong correlation with brain chronological age (Hernandez et al., 2011). Epigenetic changes by DNA methylation are generally associated with changes in gene expression, as where a greater percentage of methylation is associated with a lower gene expression, and vice-versa. An age-related loss of methylation can be explained by reduced fidelity of the maintenance methyltransferase DNMT1, whereas an age-related increase in methylation could potentially reflect the accumulation of stochastic methylation events (Christensen et al., 2009). A pangenomic analysis of age-related changes in DNA methylation showed that the DNA regions differently methylated with aging are generally associated with structural variants such as SNVs (Bell et al., 2012). It has been suggested that individuals living older than 85, and who had not developed major age-related diseases, carry more genetic or epigenetic resistant factors to sickness that attenuate the effects of disease susceptibility factors (Halaschek-Wiener et al., 2009). This was confirmed in healthy centenarians where the number of protective variants was higher than the number of genetic variants associated with diseases similar to those observed in the general population (Sebastiani and Perls, 2012).

Presently, data on combined genetic and epigenetic of complex traits in older adults are scarce even more for anxiety or depression disorders. One example is the obervation of an age-dependent. allele-specific methylation wherein young individuals (20–30 years) the difference in DNA methylation between alleles is significant, whereas in individuals older than 60 years it is not detectable (Stepanow et al., 2011). Moreover, the allele showing a decrease in methylation status was associated with an increasing body mass index and with an allele-specific transcription of the corresponding gene (Stepanow et al., 2011). In a similar approach, the objective of the present study was to evaluate, in a sample of older women, the association of anxiety disorders and/or depression with a specific DNA methylation according to SNVs in four candidate genes: SLC6A4, BDNF, OXTR, and APOE.

## Materials and Methods

### ESA Study

Participants come from the ESA Study (Enquête sur la Santé des Aînés; *Survey on Elders’ Health)*, a population-based study conducted from 2005 to 2008. The ESA Study comprises a random sample of 2811 community dwellings inhabited by French-speaking adults aged 65 years or older, living in the province of Québec, Canada (Préville et al., 2008). A random dialing method with a stratification of proportional sample of households was used, according to geographical areas (metropolitan, urban, and rural) and the 16 administrative regions of the province of Québec. A random sampling method was also used to select only one participant within the household.

Data were collected by experienced research nurses through two in-home structured interviews, separated by ∼12 months (mean = 12.5; SD = 1.4). Collected data included the French version of the Mini-Mental State Examination (Hébert et al., 1992) and the presence of DSM-IV (American Psychiatric Association, 2000) anxiety disorders (generalized anxiety disorder, panic disorder/agoraphobia, specific, phobia, and social phobia), major depressive episode and minor depression through adapted sections of the *Diagnostic Interview Schedule and Composite International Diagnostic Interview* (Robins et al., 1981; Erdman et al., 1992). The presence of anxiety disorders and depression were measured for a period of 12 months preceding each interview. Interviews were completed only with participants who scored 22 or higher on the MMSE (26 participants had a MMSE score < 22 at baseline). The research procedures of the ESA Study were previously reviewed and authorized by the ethics committee of the *Institut Universitaire de Gériatrie de Sherbrooke*. At the beginning of the interview, the participant was asked for his consent in writing to participate in the study.

### Study Sample

From the ESA Study sample, we aimed to recruit approximately 20 women who met DSM-IV criteria for anxiety disorder and/or depression in, at least, one interview and, another 20 women without anxiety or depression at both interviews, as a control group. Those with medical history of psychosis or schizophrenia were excluded. ESA participants were contacted by phone in 2010 and invited to participate in the present genetic study. Nineteen participants with previous anxiety disorder and/or depression and 24 participants without anxiety or depression (control group), were recruited. Each participant received a saliva self-sample kit (Oragene-RNA; DNA Genotek) by mail, which he had to fill according to included instructions. Instructions specified that the participants should not drink anything, except water, and should not have eaten or smoked 1 h before sampling. The saliva samples were mailed back to us through regular mail. Upon reception, samples were heated 1 h at 50°C and kept at room temperature until being processed within the following week. The present project was reviewed by the ethics committee of the *Institut Universitaire en Santé Mentale de Québec* and all participants in the study gave their written consent.

### Genetic Analyses

DNA was extracted using Qiagen columns (DNA mini kit) with a Qiacube robot (Qiagen). DNA concentrations were estimated using an intercaling fluorescent dye (Qubit), after what, DNA samples were kept at -20°C until their analyses. Targeted genes and SNVs included the apolipoprotein E (APOE) with SNVs rs429358 and rs7412, the brain derived neuropeptide factor (BDNF; rs6265), the oxytocin receptor (OXTR; rs53576) and the serotonin transporter (SLC6A4 previously named 5-HTT; 5-HTTLPR and rs25531). Selection of SNVs was made on the base of their functionality altering the functions of their encoded peptide or the expression of the gene. Except for the intronic OXTR rs53575, which was chosen because of previous association with stress reactivity (Chang et al., 2014). Genotyping of SNVs was done on a real time PCR (Lightcycler 480; Roche) using specific 5′ nuclease TaqMan assays (proprietary sequences; Life Technologies; see **Table 1** for the specific assay used). For all SNVs, PCR was made using 0.5 ul of 10X PCR Master Mix (Roche), 0.25 ul 40X TaqMan assay, 50 ng DNA in a final volume of 5 ul. PCR run included a denaturing step of 10 min 95°C followed by 35 PCR cycles (1 min 95°C, 1 min 55°C, 1 min 72°C). Length fragment polymorphism of SLC6A4 (5-HTTLPR) was determined by a specific PCR (Lotrich et al., 2008) to which was added the analysis of rs25531 (A/G; Hu et al., 2006) by MspI restriction fragment length analysis. Two non-polymorphic MspI restriction sites will cut to 312 and 356 nucleotides, respectively the 499 and 539 initial DNA fragments, that will be reduced further to 180 nucleotides in the presence of the G allele of rs25531. DNA fragments were detected by a gel electrophoresis analysis (DNA sequencer; LiCor). DNA methylation analysis was done by pyrosequencing (Pyromark 96, Qiagen) using proprietary sequences Qiagen kits (see **Table 2**), except for APOE for which pooled DNA was analyzed on Illumina Beadchips. DNA bisulfite treatment was done following manufacturer instructions (EpiTect kit; Qiagen). After purification, concentration of bisulfite treated DNA was reevaluated (Qubit).

**Table 1:** Allele frequencies for gene variants in participants with and without anxiety/depression.

Genes	Variants	Group	Alleles			*p*^1^
SLC6A4	Short S/Long L rs25531 (A/G)		Sa 312	La 356	Lg 180	
		Anxiety/depression	0.3421 (*N* = 13)	0.5526 (*N* = 21)	0.1053 (*N* = 4)	0.1601
		Control	0.4792 (*N* = 23)	0.5000 (*N* = 24)	0.0208 (*N* = 1)	
OXTR	rs53576 (G/A) Assay# C_3290335_10		G	A		
		Anxiety/depression	0.6053 (*N* = 23)	0.3947 (*N* = 15)		0.9744
		Control	0.6087 (*N* = 28)	0.3913 (*N* = 18)		
BDNF	rs6265 (C/T; Val66Met) Assay# C_11592758_10		C	T		
		Anxiety/depression	0.7667 (*N* = 23)	0.2333 (*N* = 7)		0.8897
		Control	0.7778 (*N* = 28)	0.2222 (*N* = 8)		
APOE^∗^	rs7412 (C/T; Cys112Arg) Assay# C_904973_10 rs429358 (C/T; Cys158Arg) Assay# C_3084793_20		APOE2 (TT)	APOE3 (TC)	APOE4 (CC)	
		Anxiety/depression	0.0833 (*N* = 3)	0.8333 (*N* = 30)	0.0833 (*N* = 3)	0.6785
		Control	0.0833 (*N* = 4)	0.7708 (*N* = 37)	0.1458 (*N* = 7)	

*Assay numbers (Life Technologies) are indicated when appropriate. ^∗^One anxiety subject and one control are heterozygotes CT/CT for both SNPs and their diplotype (TT, TC, or CC) could not be determined.*

*^1^*p*-value from chi-square test.*

*Number of alleles observed per group are indicated between parentheses.*

**Table 2:** Percentages of methylation among different genes forparticipants with and without anxiety/depression.

(A) Per DNA assays and CpGs.					
**Gene (PyroMark CpG assay)**	**DNA #assay**	**#CpGs**	**Anxiety/depression** **(mean ± SD) *N* = 19**	**Control** **(mean ± SD) *N* = 22**	***p***^**1**^

BDNF (Hs_BDNF_#_PM)	01	3	3.50 ± 0.85	3.19 ± 0.81	0.1158^∗^
	02	3	2.92 ± 0.74	2.34 ± 0.42	**0.0026^∗∗^**
	03	6	4.88 ± 0.67	5.26 ± 2.17	0.2232
	04	6	9.93 ± 6.47	8.25 ± 1.66	0.1414
	06	3	5.32 ± 1.23	5.08 ± 1.05	0.2414
	08	4	4.36 ± 2.55	3.58 ± 1.03	0.1220
	10	5	8.94 ± 2.55	9.44 ± 2.73	0.2747
OXTR (Hs_OXTR_#_PM)	01	4	6.40 ± 2.34	5.71 ± 2.82	0.1425^∗∗∗^
	02	5	27.45 ± 6.06	25.39 ± 8.90	0.1992
APOE (na)	01	4	0.35 ± 0.09	0.32 ± 0.10	na
SLC6A4 (Hs_SLC6A4_#_PM)	01	6	4.72 ± 0.98	4.61 ± 0.86	0.3510

^∗^*One CpG is marginally significant *p*= 0.0517.*					
^∗∗^*Wilcoxon–MannWhitney test: *p* < 0.0001.*					
^∗∗∗^*A significant difference is observed when stratifying subjects according to the SNV rs53576 (see **Table 2B**).*					
^1^**p-value resulting from *t*-tests.**					

**(B) Per SNPs and genotypes.**					

**Gene (SNP)**	**Genotypes**	**Anxiety/depression** **(mean ± SD)**	**Control (mean ± SD)**	***p***	

BDNF (rs6265 (C/T; Val66Met) DNA assay_02	CC	2.89 ± 0.89 *N* = 12	2.36 ± 0.51 *N* = 15	**0.0332^∗^**	
	CT	2.99 ± 0.41 *N* = 7	2.27 ± 0.26 N = 8	**0.0006^∗∗^**	
	TT	Na	Na	Na	
OXTR (rs53576 G/A) DNA assay_01	GG	5.93 ± 1.77 *N* = 9	6.09 ± 1.31 *N* = 8	0.4197	
	GA	5.63 ± 2.59 *N* = 5	5.69 ± 2.20 *N* = 12	0.4810	
	AA	8.02 ± 2.66 *N* = 5	4.78 ± 1.12 *N* = 3	**0.0490**	
OXTR (rs53576 G/A) DNA assay 01, CpG_2	GG	5.96 ± 1.47 *N* = 9	6.16 ± 1.33 *N* = 8	0.3851	
	GA	5.69 ± 0.92 *N* = 5	5.56 ± 1.63 *N* = 12	0.4359	
	AA	7.01 ± 0.94 *N* = 5	4.44 ± 1.11 *N* = 3	**0.0063**	

*Only those results from genes showing some differences are reported.*

*^∗^Wilcoxon–MannWhitney test: *p* = 0.0067.*

*^∗∗^Wilcoxon–MannWhitney test: *p* = 0.0065.*

*Nominally significant *p* values are in bold.*

### Statistical Analyses

Allele frequencies between affected and non-affected groups for all four targeted genes were compared using a chi-square test. Because the number of subjects is small in each group, allele frequencies should be taken with caution, thus very large differences in frequencies between the two groups are necessary to detect statistically significant results. The homo- or heteroscedasticity of the results has been tested using a *F*-test and two-tailed *t*-tests, adjusted accordingly, were used to evaluate differences between groups for mean percentages of methylation. It was shown that relatively small differences in methylation could provide statistically significant results. For example, a methylation value of 0.75 SD 0.04 observed in females, being significantly different in males (0.79 SD 0.04; *p* = 9.23e-06; Boks et al., 2009). Since the relatively small size of our sample, a Wilcoxon–MannWhitney test (SAS) was also applied. Alpha level was corrected for multiple testing according to the number of candidate genes for chi-square tests (0.05/4; adjusted *p*-value 0.0125), and to the number of methylation regions for the *t*-tests (0.05/10; adjusted *p*-value 0.005).

## Results

All 19 participants with depression had a concurrent anxiety disorder, except for one who had a major depressive disorder alone. Participants with anxiety disorder/depression did not differ from the control group (*N* = 24) according to age (mean: 70.7, SD: 5.6; control group: 72.8, 4.5; *p* = 0.200), and MMSE score results (mean: 29.4, SD: 0.8; control group: 29.1, 1.2; *p* = 0.374).

**FIGURE 1:**
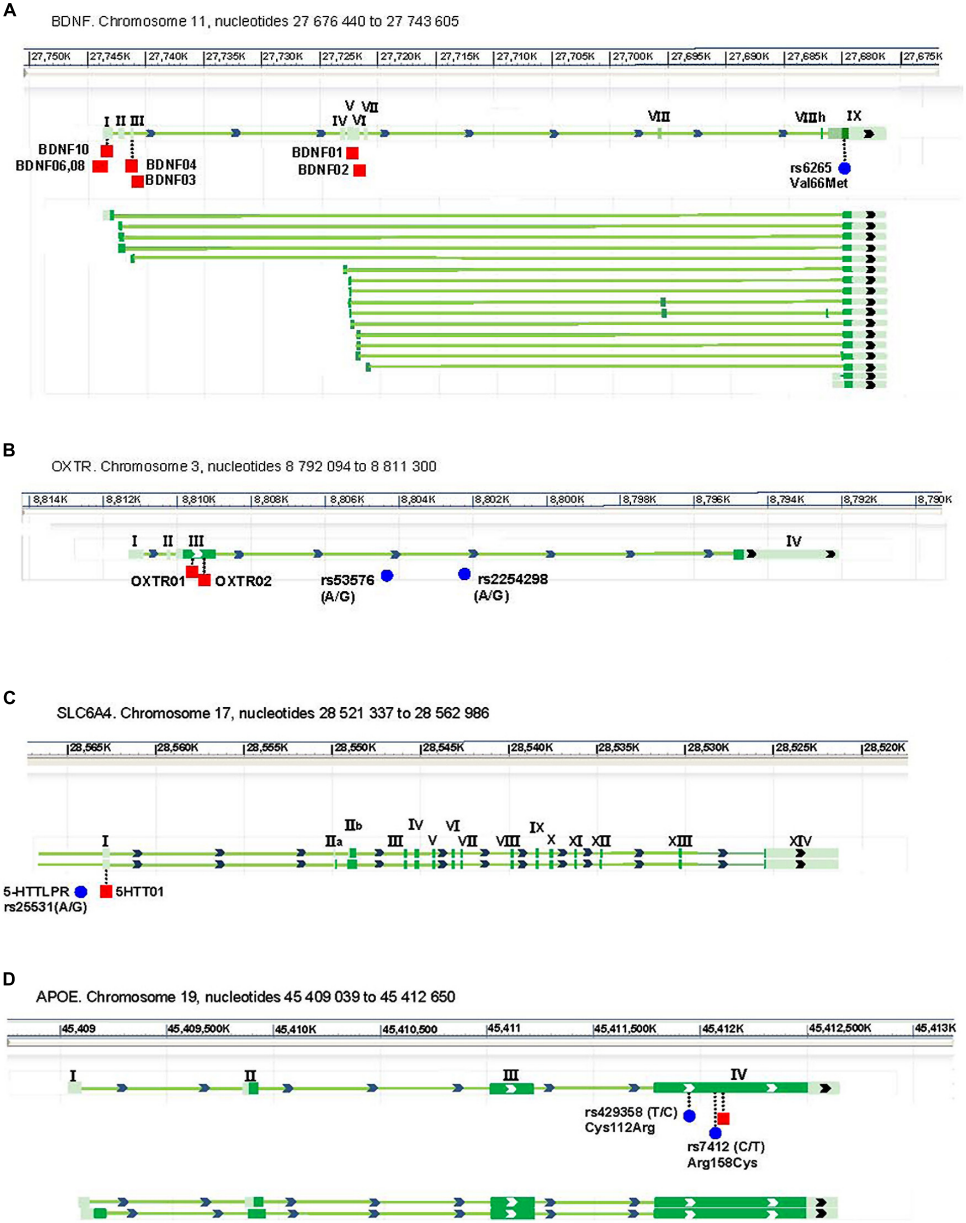
**Exon (full squares) and intron (lines) structure of BDNF **(A)**, OXTR **(B)**, SLC6A4 **(C)**, and Apoe **(D)** genes of the study, and of their different isoforms produced by differential splicing.** The location of the methylation regions (red square) and of the SNV (blue dot) are indicated where vertical lines indicate an exonic location. Exon are identified where roman letter. Adapted form NCBI database.

**Figure 1** presents the SNVs analyzed and their location in the four targeted genes of the study. For BDNF and APOE, SNVs are within coding sequences and are functional variants changing corresponding amino acids, while SLC6A4 is located in the promotor region and change level of its expression. OXTR is located in an intron and its direct effect on RNA or protein is not known. **Table 1** presents the genotyping results. No significant differences between the two groups were observed.

**Figure 1** also showed the location of the different DNA regions of the genes analyzed for their methylation. **Table 2** shows the mean methylation percentages by DNA region tested. Overall, BDNF was analyzed for seven DNA regions over 30 CpGs (3–6 CpGs per region), OXTR for two regions with nine CpGs (four and five CpGs, respectively), APOE for one region with four CpGs and SLC6A4 for one region with six CpGs. For BDNF, the percentage of methylation in one region was significantly higher in participants with anxiety/depression compared to control participants (*t*-test *p*= 0.0026; Wilcoxon–MannWhitney test *p* = 0.0001). Significant or marginally significant differences were also observed individually for all three CpGs included in this region (*t*-test *p*= 0.0439, 0.0022, and 0.0483, respectively). Moreover, this association was greater in participants with anxiety/depression carrying the CT genotype of the SNV rs6265 than in control group participants (2.99 ± 0.41 *N* = 7 vs. 2.27 ± 0.26 *N* = 8; *t*-test *p*= 0.0006; Wilcoxon–MannWhitney test *p* = 0.0065), while remaining marginally significant in participants with anxiety/depression carrying the CC genotype (2.89 ± 0.89 *N* = 12 vs. 2.36 ± 0.51 *N* = 15; *t*-test *p*= 0.0332; Wilcoxon–MannWhitney test *p* = 0.0067). No subjects carrying the TT genotype was detected in our sample.

The OXTR gene showed a higher methylation in the anxiety/depression group, but only when subjects were stratified by SNV rs53576 genotypes. Compared to the control group, only carriers of the AA genotype showed a higher percentage of methylation (near twice) in anxiety/depression participants (8.02 ± 2.66 *N* = 5 vs. 4.78 ± 1.12 *N* = 3; *p*= 0.0490) while no significant differences was observed for the two other genotypes AG (5.63 ± 2.59 *N* = 5 vs. 5.69 ± 2.20 *N* = 12; *p*= 0.4810) and GG (5.93 ± 1.77 *N* = 9 vs. 6.09 ± 1.31 *N* = 8, *p*= 0.4197; **Table 2B**). The significant difference observed came mainly from CpG 2 of the four CpGs contained in this region (7.01 ± 0.94 vs. 4.44 ± 1.11; *p*= 0.0063; **Table 2B**), the other three CpGs giving non-significant results (0.06 < *p* < 0.10). However, the OXTR results became non-significant when using the Wilcoxon–MannWhitney test. APOE did not show great differences when compared over all four CpGs (**Table 2**), while no association was observed for the gene SLC6A4.

## Discussion

The objectives of the present study were to verify whether older women with anxiety disorder/depression differ from control subjects according to DNA methylation and genotypes at SNVs in BDNF, OXTR, SLC6A4, and APOE. The results indicated that older women with anxiety disorders/depression differed on BDNF DNA methylation level from the women in the control group, particularly for the CT carriers. Moreover, the higher DNA methylation in BDNF CT genotype carriers was confirmed in a second group of women with anxiety/depression disorders and healthy controls (3.36 ± 0.15 *N* = 4 vs. 2.93 ± 0.08 *N* = 4; *t*-test *p* = 0.0022), while CC and TT carriers (*N* = 4 each) showed no significant differences. On the other hand, groups also differed on OXTR DNA methylation level, but this effect was dependent to specific OXTR SNVs and disappeared on the Wilcoxon–MannWhitney test.

Previous results suggested that a lower level of BDNF expression is associated with depression (Groves, 2007). This was observed in human with depression (Marvanova et al., 2001; Karege et al., 2002, 2005; Dwivedi et al., 2003; Aydemir et al., 2005) as well as in a mice model of depression (Tsankova et al., 2006). In this study we observed a higher methylation of BDNF in older women with anxiety disorders/depression. Traditionally, DNA methylation has been thought of as being involved in gene silencing, even if research in recent years has shown a more complex picture (Muers, 2013). One to 10% differences in methylation were reported to be significantly associated to gene expression (Gervin et al., 2012). We would need to evaluate BDNF RNA expression to confirm this possibility. If so, we will have to take into account the isoforms of BDNF as shown in mice results where only two of the five BDNF isoforms tested showed a different expression in depressive mice (Tsankova et al., 2006). In human, BDNF showed a high number of isoforms (see **Figure 1**) that could be highly regulated in their expression according to biological (tissue, age, sex) or environmental (stress, pollution, nutrition, medication) factors. Moreover, these isoforms could have significant different phenotypical properties that could be opposite in effect. For example, the short transcript form of the BCL-X gene promotes apoptosis, while the long form inhibits cell death (Boise et al., 1993).

DNA methylation is naturally greater in CpG rich islands of older persons (Horvath, 2013). Since we compared subjects with anxiety-disorders/depression to control group subjects of the same age group, higher DNA methylation in subjects with anxiety disorders/depression did not come from aging *per se*. Similarly, sex, comorbidities and ethnicity did not come into play since all individuals were female of caucasian origins with no other obvious disease.

The OXTR gene has been less studied in relation to anxiety/depression. One study indicated an association between the GG genotype of the SNV rs53576 with higher levels of adult separation anxiety (Costa et al., 2009). We observed in older adults a possible higher DNA methylation in the anxiety disorders/depression group for carriers of the AA genotype compared to the control group. This finding contrasts with previous results showing an association between anxiety and GG genotype in younger adults. A higher DNA methylation of AA subjects is probably associated with a lower OXTR expression. We can hypothesize that for OXTR the effects of the different genotypes of SNV rs53576 varied according to age (adolescent vs. young adult vs. old adults), as observed previously with body mass index (Stepanow et al., 2011), since etiologies of mental diseases may differ between old and young adults. Previous results indicated a different genetic influence between young and old adults on neuropathological manifestations associated with depression (Taylor et al., 2005). Genetic predispositions to mental diseases are modulated by environmental factors and their effects could cumulate through aging by the addition of stressful events endured during life (Lupien et al., 2005). Previous studies observed an association between the BDNF Met allele in older adults (Pei et al., 2012), but not in younger adults (Verhagen et al., 2010), and it was proposed that a decreased activity in the BDNF system may have a stronger effect in older adults (Pei et al., 2012). Similarly, the short form of 5-HTTLPR polymorphism of SLC6A4 was associated with reduced hippocampal volume in older adults with early onset depression (at a younger age) whereas the long form was associated with reduce hippocampal volume in older adults with late-onset depression (Taylor et al., 2005). For instance, depression arising at an advanced age could be due to prodromal Alzheimer’s disease (Panza et al., 2010), cerebrovascular pathology (Alexopoulos et al., 1997; Alexopoulos, 2006), or subcortical Lewy bodies (Tsopelas et al., 2011).

The present study is one of a few to examine DNA methylation in older adults with anxiety disorders/depression. The results have to be interpreted in light of some limitations. First, the epigenetic measures were obtained from peripheral tissue (saliva), which raises the problem of brain concordance of the results. Although imperfect, it is now recognized that peripheral tissues such as blood or saliva (Horvath et al., 2012; Thompson et al., 2013) could be good surrogates for brain tissue when studying DNA methylation or RNA expression. Secondly, saliva was not sampled at the same time that mental health evaluation was done. Therefore, it is possible that the DNA methylation level did not reflect exactly the initial anxiety/depressive symptoms. And finally, the present sample is relatively modest and future studies using larger samples would be required to confirm our findings.

These preliminary results suggest that genetic (SNV) and epigenetic (DNA methylation) factors interactions in BDNF and eventually in OXTR genes are involved in the anxiety disorders/depression phenotype in older adults. However, it is expected that these two genes alone do not explain all cases of anxiety disorders/depression in older women, and that additional genes could be involved. Overall, the present preliminary findings need to be confirmed in women, and men should also be investigated in future studies.

## Conflict of Interest Statement

The authors declare that the research was conducted in the absence of any commercial or financial relationships that could be construed as a potential conflict of interest.
