# Supplementary material for: DNA methylation and single nucleotide variants in the brain-derived neurotrophic factor (BDNF) and oxytocin receptor (OXTR) genes are associated with anxiety/depression in older women
Source: Front Genet. 2015 Jun 30;6:230. doi: 10.3389/fgene.2015.00230 (PMC4485183; doi:10.3389/fgene.2015.00230)
Supplement: Supplementary file 1 [file Data_Sheet_1.PDF]

Annexe 1. DSM-IV criteria to diagnose subjects of the study.

Panic disorder without agoraphobia

A. Both (1) and (2):
   (1) recurrent unexpected panic attacks (see p. 432)
   (2) at least one of the attacks has been followed by 1 month (or more) of one (or more) of the following:
      (a) persistent concern about having additional attacks
      (b) worry about the implications of the attack or its consequences (e.g., losing control, having a heart attack, "going crazy")
      (c) a significant change in behavior related to the attacks

B. Absence of agoraphobia (see p. 433) .

C. The panic attacks are not due to the direct physiological effects of a substance (e.g.,a drug of abuse, a medication) or a general medical condition (e.g., hyperthyroidism).

D. The panic attacks are not better accounted for by another mental disorder, such associal phobia (e.g., occurring on exposure to feared social situations), specific phobia (e.g., onexposure to a specific phobic situation). Obsessive-compulsive disorder(e.g., on exposure to dirt in someone with an obsession about contamination), posttraumatic stress disorder (e.g., in response to stimuli associated with a severe stressor), or separation anxiety disorder (e.g., in response to being away from home orclose relatives).

Agoraphobia without history of panic disorder

A. The presence of agoraphobia (see p. 433) related to fear of developing panic-like symptoms (e.g., dizziness or diarrhea).

B. Criteria have never been met for panic disorder (see p. 440).

C. The disturbance is not due to the direct physiological effects of a substance(e.g., a drug of abuse,a medication) or a general medical condition.

D. If an associated general medical condition is present. The fear described in criterion a is clearly in excess of that usually associated with the condition

Specific phobia

A. Marked and persistent fear that is excessive or unreasonable, cued by the presence or anticipation of a specific object or situation (e.g., flying, heights, animals, receiving an injection, seeing blood).

B. Exposure to the phobic stimulus almost invariably provokes an immediate anxiety response, which may take the form of a situationally bound or situationally predisposed panic attack. Note: in children, the anxiety may be expressed by crying,tantrums, freezing, or clinging.

C. The person recognizes that the fear is excessive or unreasonable. Note: in children, this feature may be absent.

D. The phobic situation(s) is avoided or else is endured with intense anxiety or distress.

E. The avoidance, anxious anticipation, or distress in the feared situation(s) interferes significantly with the person's normal routine, occupational (or academic) functioning, or

social activities or relationships, or there is marked distress about having the phobia.

F. In individuals under age 18 years, the duration is at least 6 months.

G. The anxiety, panic attacks, or phobic avoidance associated with the specific object or situation are not better accounted for by another mental disorder, such as obsessive-compulsive disorder (e.g., fear of dirt in someone with an obsession about contamination), posttraumatic stress disorder (e.g., avoidance of stimuli associated with asevere stressor), separation anxiety disorder (e.g., avoidance of school), social phobia (e.g., avoidance of social situations because offear of embarrassment), panic disorder with agoraphobia, or agoraphobia without history of panic disorder.

Social phobia

A. A marked and persistent fear of one or more social or performance situations in which the person is exposed to unfamiliar people or to possible scrutiny by others. The individual fears that he or she will act in a way (or show anxiety symptoms) that will be humiliating or embarrassing. Note: in children, there must be evidence of the capacity for age-appropriate social relationships with familiar people and the anxiety must occur in peer settings, not just in interactions with adults.

B. Exposure to the feared social situation almost invariably provokes anxiety, which may take the form of a situationally bound or situationally predisposed panic attack. Note: in children, the anxiety may be expressed by crying, tantrums, freezing, or shrinking from social situations with unfamiliar people.

C. The person recognizes that the fear is excessive or unreasonable. Note: in children, this feature may be absent.

D. The feared social or performance situations are avoided or else are endured with intense anxiety or distress.

E. The avoidance, anxious anticipation, or distress in the feared social or performance situation(s) interferes significantly with the person's normal routine, occupational(academic) functioning, or social activities or relationships, or there is marked distress about having the phobia.

F. In individuals under age 18 years, the duration is at least 6 months.

G. The fear or avoidance is not due to the direct physiological effects of a substance(e.g., a drug of abuse. A medication) or a general medical condition and is not better accounted for by another mental disorder (e.g., panic disorder with or without agoraphobia. Separation anxiety disorder, body dysmorphic disorder, a pervasivedevelopmental disorder, or schizoid personality disorder).

H. If a genera l medical condition or another mental disorder is present, the fear in criterion a is unrelated to it, e .g., the fear is not of stuttering, trembling in Parkinson'sdisease, or exhibiting abnormal eating behavior in anorexia nervosa or bulimia nervosa.

Generalized anxiety disorder

A. Excessive anxiety and worry (apprehensive expectation), occurring more days thannot for at least 6 months, about a number of events or activities (such as work orschool performance).

B. The person finds it difficult to control the worry.

C. The anxiety and worry are associated with three (or more) of the following six symptoms (with at least some symptoms present for more days than not for the past6 months), note: only one item is required in children.

(1) restlessness or feeling keyed up or on edge
(2) being easily fatigued
(3) difficulty concentrating or mind going blank
(4) irritability
(5) muscle tension
(6) sleep disturbance (difficulty falling or staying asleep, or restless unsatisfying sleep)

D. The focus of the anxiety and worry is not confined to features of an axis I disorder, e.g ., the anxiety or worry is not about having a panic attack (as in panic disorder),being embarrassed in public (as in social phobia), being contaminated (as in obsessive-compulsive disorder), being away from home or close relatives (as in separationanxiety disorder), gaining weight (as in anorexia nervosa), having multiple physical complaints (as in somatization disorder), or having a serious illness (as in hypochondriasis), and the anxiety and worry do not occur exclusively during posttraumaticstress disorder.

E. The anxiety, worry, or physical symptoms cause clinically significant distress or impairment in social, occupational, or other important areas of functioning.

F. The disturbance is not due to the direct physiological effects of a substance (e.g., a drug of abuse, a medication) or a general medical condition (e. G., hyperthyroidism) and does not occur exclusively during a mood disorder, a psychotic disorder, o r a pervasive developmental disorder

Major depressive episode

A. Five (or more) of the following symptoms have been present during the same 2-week period and represent a change from previous functioning; at least one of the symptoms is either (1) depressed mood or (2) loss of interest or pleasure.
Note: do not include symptoms that are clearly due to a general medical condition, or mood-incongruent delusions or hallucinations.

(1) depressed mood most of the day, nearly every day, as indicated by either subjective report (e.g., feels sad or empty) or observation made by others (e.g., appears tearful). Note: in children and adolescents, can be irritable mood.
(2) Markedly diminished interest or pleasure in all, or almost all, activities most of the day, nearly every day (as indicated by either subjective account or observation made by others)
(3) significant weight loss when not dieting or weight gain (e.g., a change of morethan 5% of body weight in a month), or decrease or increase in appetite nearlyevery day. Note: in children, consider failure to make expected weight gains.

(4) insomnia or hypersomnia nearly every day
(5) psychomotor agitation or retardation nearly every day (observable by others,not merely subjective feelings of restlessness or being slowed down)
(6) fatigue or loss of energy nearly every day
(7) feelings of worthlessness or excessive or inappropriate guilt (which may be delusional) nearly every day (not merely self-reproach or guilt about being sick)
(8) diminished ability to think or concentrate, or indecisiveness, nearly every day(either by subjective account or as observed by others)
(9) recurrent thoughts of death (not just fear of dying), recurrent suicidal ideationwithout a specific plan, or a suicide attempt or a specific plan for committingsuicide

B. The symptoms do not meet criteria for a mixed episode (see p. 365).
C. The symptoms cause clinically significant distress or impairment in social, occupational, or other important areas of functioning.
D. The symptoms are not due to the direct physiological effects of a substance (e.g., a drug of abuse, a medication) or a general medical condition (e.g., hypothyroidism).
E. The symptoms are not better accounted for by bereavement, i .e., after the loss of aloved one, the symptoms persist for longer than 2 months or are characterized bymarked functional impairment, morbid preoccupation with worthlessness, suicidalideation, psychotic symptoms, or psychomotor retardation.

Minor depressive disorder

A. A mood disturbance, defined as follows:

(1) at least two (but less than five) of the following symptoms have been present during the same 2-week period and represent a change from previous functioning; at least one of the symptom s is either (A) or (B):

(a) depressed mood most of the day, nearly every day, as indicated by either subjective report (e.g., feels sad or empty) or observation made by others(e.g., appears tearful). Note: in children and adolescents, can be irritablemood.
(b) markedly diminished interest or pleasure in all, or almost all, activities mostof the day, nearly every day (as indicated by either subjective account orobservation made by others)
(c) significant weight loss when not dieting or weight gain (e.g., a change ofmore than 5% of body weight in a month), or decrease or increase in appetite nearly every day. Note: in children, consider failure to make expected weight gains.
(d) insomnia or hypersomnia nearly every day
(e) psychomotor agitation or retardation nearly every day (observable by others, not merely subjective fee lings of restlessness or being slowed down)
(f) fatigue or loss of energy nearly every day
(g) feelings of worthlessness or excessive or inappropriate guilt (which may be delusional) nearly every day (not merely self-reproach or guilt about beingsick)
(h) diminished ability to think or concentrate, or indecisiveness, nearly every day (either by subjective account or as observed by others)

(i) recurrent thoughts of death (not just fear of dying), recurrent suicidal ideation without a specific plan, or a suicide attempt or a specific plan for committing suicide

(2) the symptoms cause clinically significant di stress or impairment in social, occupational, or other important areas of functioning

(3) the symptomsare not due to the direct physiological effects of a substance (e.g.a drug of abuse, a medication) or a general medical condition (e.g., hypothyroidism)

(4) the symptoms are not better accounted for by bereavement (i.e. a normal reaction to the death of a loved one)

B. There has never been a major depressive episode (see p. 356), and criteria are notmet for dysthymic disorder.

C. There has never been a manic episode (see p. 362), a mixed episode (see p. 365), ora hypomanic episode (see p. 368). And criteria are not met for cyclothymicdisorder. Note: this exclusion does not apply if all of the manic-, mixed -, or hypomanic-like episodes are substance or treatment induced.

D. The mood disturbance does not occur exclusively during schizophrenia, schizophreniform disorder. Schizoaffective disorder, delusional disorder, or psychotic disorder not otherwise specified.
